# The Influence of Nitrogen on the Biological Properties of Soil Contaminated with Zinc

**DOI:** 10.1007/s00128-016-1977-2

**Published:** 2016-11-21

**Authors:** Rafał Strachel, Jadwiga Wyszkowska, Małgorzata Baćmaga

**Affiliations:** Department of Microbiology, University of Warmia and Mazury, Plac Łódzki 3, 10-727 Olsztyn, Poland

**Keywords:** Zinc, Soil, Hydrolases, Microorganisms, Nitrogen, Biostimulation

## Abstract

This study analyzed the relationship between nitrogen fertilization and the biological properties of soil contaminated with zinc. The influence of various concentrations of zinc and nitrogen on the microbiological and biochemical activity of soil was investigated. In a laboratory experiment, loamy sand with pH_KCl_ 5.6 was contaminated with zinc (ZnCl_2_) and fertilized with urea as a source of nitrogen. The activity of acid phosphatase, alkaline phosphatase, urease and β-glucosidase, and microbial counts were determined in soil samples after 2 and 20 weeks of incubation. Zinc generally stimulated hydrolase activity, but the highest zinc dose (1250 mg kg^−1^) led to the inhibition of hydrolases. Nitrogen was not highly effective in neutralizing zinc’s negative effect on enzyme activity, but it stimulated the growth of soil-dwelling microorganisms. The changes in soil acidity observed after the addition of urea modified the structure of microbial communities.

Soil is a crucial element of the biosphere, and it plays a very important role in agricultural production. Toxic compounds are neutralized in the soil environment where various biological processes enhance the biogeochemical cycling of elements (Vrščaj et al. 2008). Most of those processes involve microorganisms that immobilize biogenic elements such as carbon, nitrogen and phosphorus. Soil microbiological consortia play a key role in the cycling of substances in nature. Excessive accumulation of toxic substances, such as heavy metals, disrupts microbial development (Adriano et al. 2004; Knopf and König 2010; Strachel et al. 2016). Since the beginning of the industrial revolution, pollution with heavy metals has accelerated dramatically, posing a substantial risk for ecosystem health, in particular in agricultural production (Jing et al. 2007). Zinc is less toxic than other heavy metals (Cd, Hg, Pb, As), but excessive accumulation of this element in the soil ecosystem can exert a negative influence on soil microorganisms which are the main determinants of soil fertility (McLaughlin et al. 1999). Zinc contamination decreases the quantity and quality of crops grown in areas polluted with this metal (Chibuike and Obiora 2014; Yang et al. 2006; Wyszkowska et al. 2016). Nitrogen fertilization significantly improves the availability of nitrogen in soil, and it stimulates the growth of soil-dwelling microorganisms. The microbiological diversity of soil is conditioned by nutrient availability, and it is significantly influenced by fertilization (Wang et al. 2015). The biochemical activity of soil is a reliable indicator of fertility, and it can change under exposure to toxic substances (Chaperon and Sauvé 2007; Kızılkaya et al. 2004). Hydrolase activity, a robust measure of soil health, is influenced by the counts, composition and enzymatic potential of soil-dwelling microorganisms, exposure to protective treatments or substances that inhibit enzyme activity. This study evaluated the effects of nitrogen fertilization in zinc-contaminated soil on the activity of selected hydrolases: acid phosphatase, alkaline phosphatase, urease and β-glucosidase, and on the counts of selected groups of soil-dwelling microorganisms. Those enzymes are reliable indicators of soil quality because they participate directly in the cycling of elements (carbon, nitrogen and phosphorus) and respond differently to stressors, including zinc. In the present study, urea was applied as the only source of nitrogen to evaluate its biostimulatory effect on soil enzymes and microorganisms.

## Materials and Methods

Soil samples for the experiment were obtained from the Agricultural Experiment Station in Tomaszkowo (north-eastern Poland, 53.71610 N, 20.41670 E) operated by the University of Warmia and Mazury in Olsztyn. Samples collected from the arable layer (depth of 0–20 cm) had the granulometric composition of loamy sand and were classified as Eutric Cambisols according to the World Reference Base of Soil Resources (2014). Before the experiment, soil was thoroughly mixed and selected physicochemical properties of soil were determined. Soil samples had the following parameters: pH (in 1 Mol KCl dm^−3^) – 5.6 ± 0.2, C_org_ – 10.0 ± 3.2 g kg^−1^ soil DM, N_total_ – 0.58 ± 0,07 g kg^−1^ soil DM, available cations (mg kg^−1^ soil DM): P – 96.32 ± 13.48, K – 179.08 ± 35.82, Mg – 50.17 ± 8.03; total exchangeable cations (mg kg^−1^ soil DM): K^+^ – 217.73, Ca^2+^ – 568.60, Na^+^ – 100.34, Mg^2+^ – 64.52; hydrolytic acidity (HAC) – 18.66 ± 0.43 mMol^(+)^ kg^−1^ soil DM; total exchangeable bases (TEB) – 40.00 ± 2.0 mMol^(+)^ kg^−1^ soil DM; cation exchange capacity (CEC) – 58.66 ± 1.64 mM(+) kg^−1^ soil DM; base saturation (BS) – 68.19% ± 1.55; Zn _total_ – 22.68 ± 2.72 mg kg^−1^ DM soil; Zn _bioavailable_ – 9.13 ± 1.19 mg kg^−1^ soil DM.

The experiment was carried out in 150 cm^3^ glass beakers. A different set of beakers was used for every analytical date, which produced a total of 108 beakers (six zinc doses × three nitrogen doses × two analytical dates × three replications). Soil samples of 100 g (particle size ≤2.0 mm) were combined with ZnCl_2_ in doses of 0, 250, 500, 750, 1000 and 1250 mg Zn^2+^ kg^−1^ soil DM. Nitrogen’s influence on the biological properties of zinc-contaminated soil was determined by fertilizing soil samples with urea at the rate of 0, 250 and 500 mg N kg^−1^ soil DM. Soil samples were incubated for 2 and 20 weeks at a temperature of 25°C and at 50% capillary water capacity. After 2 and 20 weeks of incubation, the activity of acid phosphatase, alkaline phosphatase, urease and β-glucosidase was determined colorimetrically by the method proposed by Alef and Nannipieri (1998). The analyses were carried out in triplicate. The following substrates were used in enzyme activity tests: 4-nitrophenyl phosphate disodium (for acid phosphatase and alkaline phosphatase, λ = 410 nm), 10% urea solution (for urease, λ = 410 nm), and p*-*nitrophenyl*-*β*-*D*-*glucopyranoside (for β-glucosidase, λ = 400). The activity of acid phosphatase, alkaline phosphatase and β-glucosidase was expressed in mmol 4-nitrophenol (PNP) kg^−1^ soil DM h^−1^, and the activity of urease – in mmol N-NH_4_ kg^−1^ soil DM h^−1^. The analyses were performed with the use of the Cecil Aquarius 7500 spectrophotometer with a wavelength range of 190–1100 nm and accuracy of 0.5 nm. The accuracy of measurements was as follows: activity of acid phosphatase and alkaline phosphatase – 0.0004 µM PNP, activity of β-glucosidase – 0.0001 µM PNP, activity of urease – 0.0010 mM N-NH_4_. Microbial counts were determined by the streak plate method in three replications. Organotrophic bacteria were cultured on soil extract with the addition of Bunt and Rovira’s medium (1955), and oligotrophic and copiotrophic bacteria were grown on substrate containing peptone and meat extract (Onta and Hattori 1983). Actinomycetes were cultured on Küster and Williams’ medium with the addition of antibiotics nystatin and actidione (Parkinson et al. 1971). Fungal counts were determined on glucose peptone agar with the addition of rose bengal and aureomycin according to Martin’s method (1950). Selected physicochemical parameters of soil were analyzed after 20 weeks of incubation (in triplicate). Soil pH was determined potentiometrically in 1 M KCl aqueous solution; hydrolytic acidity (HAC), total exchangeable bases (TEB), cation exchange capacity (CEC) and base saturation (BS) were determined according to the methods described by Carter (1993). Soil samples (after 20 weeks incubation) were assayed for bioavailable zinc and total zinc content by flame atomic absorption spectrometry (FAAS). Zinc concentration in soil was determined with the use of Metranal® 32 Quality Control Material (QCM). The limit of detection for FAAS was 96 µg dm^−3^. The total zinc content in soil was determined with the use of Atomic Absorption Spectrometr AAS 30, and bioavailable zinc content – Atomic Absorption Spectrometr GBC 932 AA. The results were analyzed statistically in the Statistica 12 program (StatSoft Inc. 2014). Homogeneous groups were identified in Tukey’s test at a significance level of *p* = 0.01. The proportion of variance associated with the observed effects (η^2^) was determined, principal component analysis (PCA) was performed, and the between the degree of soil contamination with zinc, enzyme activity, microbial counts and physicochemical properties of soil were computed. The formula proposed by Kaczyńska et al. (2015) was used to calculate the biostimulation index (IF_b_): IF_b_ = A_b_/A, where: A_b_ – enzyme activity or microbial counts in soil with urea, A – enzyme activity or microbial counts in soil without urea.

## Results and Discussion

In this experiment, enzyme activity was influenced mainly by the applied dose of zinc (*p* ≤ 0.001) (Table 1). Urease activity was most (46.67%) influenced by an interaction between two experimental factors (zinc dose x soil incubation time) (*p* ≤ 0.001). Urea influenced acid phosphatase, whereas incubation time significantly affected the activity of β-glucosidase and, to a lesser extent, the activity of alkaline phosphatase.

**Table 1:** Proportion of variance η^2^ observed in soil contaminated with zinc

Variables	Pac	Pal	Ure	Glu
Dose of Zn	51.04	42.63	2.62	41.84
Dose of N	26.40	1.76	3.74	1.22
Incubation time	0.43	12.87	0.42	35.10
Dose of Zn · dose of N	10.39	22.50	14.51	10.46
Dose of Zn · incubation time	2.45	5.44	46.67	3.11
Dose of N · incubation time	6.49	4.96	0.59	1.64
Dose of Zn · dose of N · incubation time	2.63	9.34	29.97	6.41
Error	0.16	0.51	1.48	0.21

*Zn* zinc, *N* nitrogen, *Pac* acid phosphatase, *Pal* alkaline phosphatase, *Ure* urease, *Glu* β-glucosidase

**Fig. 1:**
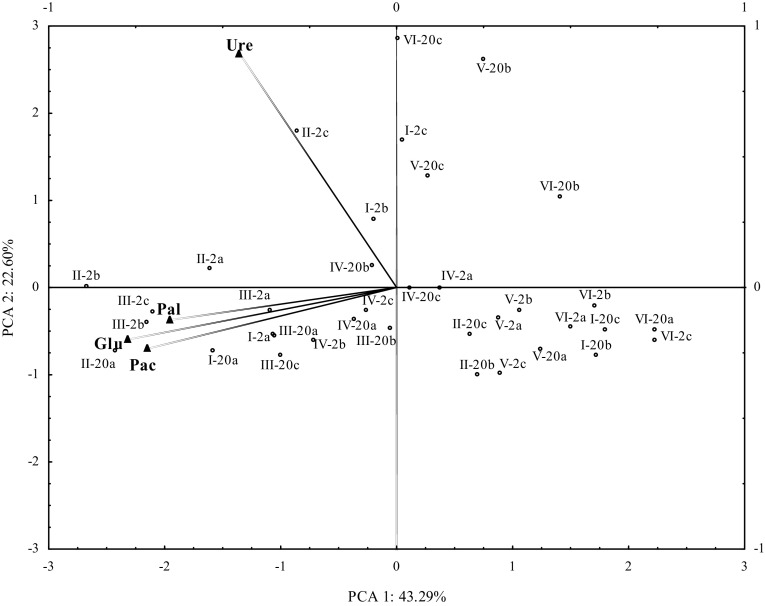
Hydrolase activity in soil contaminated with zinc. *Pac* acid phosphatase, *Pal* alkaline phosphatase, *Ure* urease, *Glu* β-glucosidase; dose of Zn^2+^ (mg kg^−1^): *I* – 0, *II* – 250, *III* – 500, *IV* – 750, *V* – 1000, *VI* – 1250; incubation time: 2–2 weeks, 20–20 weeks; dose of N (mg kg^−1^): *a* – 0, *b* – 250, *c* – 500

The distribution of vectors describing enzyme activity (PCA) revealed significant correlations between enzyme activity and the applied dose of zinc (Fig. 1). Zinc doses of 250 and 500 mg Zn^2+^ kg^−1^ stimulated enzyme activity, whereas higher doses of this element had an inhibitory effect on soil enzymes. This is illustrated by the distribution of cases on a plane. Zinc’s inhibitory influence on the biochemical activity of soil was ambiguous. In a study by Yang et al. (2006), zinc doses of 100 to 800 mg kg^−1^ led to a linear reduction in urease activity and an increase in alkaline phosphatase activity. In the work of Kucharski et al. (2011), the negative consequences of zinc contamination were exacerbated with an increase in dose, and zinc’s inhibitory effect persisted for 120 days. Enzymes were arranged in the following order based on their resistance to zinc: β-glucosidase > urease > acid phosphatase. A similar relationship was noted by Borowik et al. (2014b) where a zinc dose of 2400 mg Zn^2+^ kg^−1^ inhibited enzyme activity in the following order: alkaline phosphatase > urease > acid phosphatase > β-glucosidase. In the present study, the analyzed enzymes were arranged in the following order based on their sensitivity to a zinc dose of 1250 mg Zn^2+^ kg^−1^: urease > acid phosphatase > alkaline phosphatase > β-glucosidase. β-glucosidase was least susceptible to all doses of zinc, but according to Kucharski et al. (2011), the enzyme’s resistance decreases under exposure to higher soil acidity.

Acid phosphatase, alkaline phosphatase and β-glucosidase responded similarly to soil contamination with zinc, which is demonstrated by the proximity of vectors representing the analyzed enzymes. Urease was also sensitive to zinc, but its response to this heavy metal was somewhat different. This is illustrated by the position of the urease vector relative to cases representing the highest zinc doses. The neutralizing effects of nitrogen fertilizer on the biochemical activity of zinc- contaminated soil were observed after 2 weeks of incubation. Neutralizing effects were not noted after 20 weeks of soil incubation. Our findings suggest that enzyme activity is influenced by environmental and anthropogenic factors and by the enzyme’s specific properties.

The zinc content of soil, determined by FAAS, increased linearly. Total zinc concentrations ranged from 23 mg kg^−1^ DM soil in uncontaminated treatments to 1100 mg kg^−1^ DM soil in the most contaminated treatments. The respective values for bioavailable zinc were 8.3–1034 mg kg^−1^ DM soil. Similarly to the nominal zinc content, the above results correlated with the microbiological, biochemical and physicochemical properties of soil. Linear correlation coefficients revealed significant negative correlations between Zn^2+^ vs. acid phosphatase activity (*p* ≤ 0.001), β-glucosidase activity (*p* = 0.004), counts of copiotrophic (*p* = 0.034) and oligotrophic bacteria (*p* ≤ 0.001) and Actinomycetes counts (*p* = 0.004) (Table 2). There was a highly significant positive correlation between zinc dose vs. urease activity (*p* ≤ 0.001) and fungal counts (*p* ≤ 0.001). Soil reaction significantly influences the biochemical properties of soil contaminated with heavy metals, and the introduction of urea to the soil ecosystem can decrease soil pH. The above was also observed in this study. The introduction of urea to soil not contaminated with zinc led to a decrease in soil pH (Table 3). The above relations can be attributed to the nitrification of ammonium ions produced during urea hydrolysis, which changes the solubility of heavy metals. Changes in soil pH can also compromise microbial growth (Geisseler and Scow 2014). Zinc contamination of soil fertilized and not fertilized with urea led to changes in soil pH. In treatments without urea, soil acidity increased with an increase in zinc dose. In treatments fertilized with nitrogen, zinc limited soil acidity. The above can probably be attributed to zinc’s inhibitory influence on nitrification. In a study by Borowik et al. (2014a), zinc contamination of soil limited the development of nitrifying bacteria and disrupted nitrification. The nitrification process was effectively inhibited already by a dose of 300 mg Zn^2+^ kg^−1^ DM soil.

In this study, the application of urea fertilizer lowered soil pH and increased hydrolytic acidity which was significantly negatively correlated with the activity of all tested enzymes (Pac, Ure, Glu: *p* ≤ 0.001; Pal: *p* = 0.03) and the counts of the examined microbial groups (Cop: *p* = 0.033; Olig: *p* = 0.038; Act: *p* ≤ 0.001; Fun: *p* = 0.011), excluding organotrophic bacteria (*p* = 0.085).

**Table 2:** Correlation coefficients demonstrating the strength of relationships between the physicochemical properties of soil vs. enzyme activity and microbial counts after 20 weeks of incubation

	Zn_T_	Zn_B_	Pac	Pal	Ure	Glu	Org	Cop	Olig	Act	Fun
Pac	−0.62**	−0.62**									
Pal	−0.05	−0.05	0.31*								
Ure	0.51**	0.51**	−0.23	0.06							
Glu	−0.37**	−0.37**	0.72**	0.64**	0.11						
Org	0.08	0.08	0.11	0.58**	0.41**	0.35**					
Cop	−0.27*	−0.28*	0.06	0.36**	−0.20	0.34*	0.23				
Olig	−0.57**	−0.58**	0.29*	0.44**	−0.21	0.38**	0.41**	0.68**			
Act	−0.38**	−0.39**	0.84**	0.34*	0.02	0.74**	0.22	0.00	0.17		
Fun	0.85**	0.85**	−0.55**	0.23	0.55**	−0.16	0.24	−0.12	−0.42**	−0.31*	
pH	0.21	0.21	0.55**	0.23	0.12	0.39**	0.02	−0.32*	−0.36**	0.62**	0.17
HAC	−0.30*	−0.30*	−0.43**	−0.30*	−0.43**	−0.46**	−0.24	0.29*	0.28*	−0.64**	−0.34*
TEB	0.30*	0.30*	0.24	0.36**	0.71**	0.51**	0.51**	−0.12	−0.04	0.52**	0.33*
CEC	0.27*	0.27*	0.17	0.35**	0.74**	0.48**	0.55**	−0.07	0.03	0.46**	0.30*
BS	0.34*	0.34*	0.26	0.44**	0.68**	0.55**	0.49**	−0.07	−0.05	0.53**	0.40**

*Zn*_*T*_ total zinc content, *Zn*_*B*_ bioavailable zinc content, *Pac* acid phosphatase, *Pal* alkaline phosphatase, *Ure* urease, *Glu* β-glucosidase, *Org* organotrophic bacteria, *Cop* copiotrophic bacteria, *Olig* oligotrophic bacteria, *Act* actinomycetes, *Fun* fungi, *HAC* hydrolytic acidity, *TEB* total exchangeable bases, *CEC* cation exchange capacity, *BS* base saturation (*p = 0.05, **p = 0.01; n = 53)

Nitrogen compounds present in soil are transformed mainly by microorganisms. The content of C and the C:N ratio influence microbial growth (Booth et al. 2005). Taylor et al. (2002) revealed strong correlations between organic matter content, microbial counts and enzyme activity. According to Moreno et al. (2009), the activity of urease, acid phosphatase and β-glucosidase in zinc-contaminated soil decreased with an increase in zinc dose, but zinc stimulated enzyme activity in soils with a high content of organic matter. Those observations can be largely attributed to the protective role of organic matter in stabilizing the biochemical properties of soil (Wyszkowska et al. 2015). In the present study, the effects of urea on the biochemical parameters of soil were evaluated in view of nitrogen’s ability to neutralize zinc in soils with a narrow C:N ratio.

**Table 3:** Soil pH after 20 weeks of incubation

Dose of zinc (mg Zn^2+^ kg^−1^)	Dose of nitrogen (mg N kg^−1^)		
	0	250	500
0	5.517 ± 0.029^a^	4.800 ± 0.001^ef^	4.767 ± 0.058^f^
250	5.500 ± 0.001^ab^	4.900 ± 0.001^def^	4.800 ± 0.001^ef^
500	5.500 ± 0.001^ab^	4.983 ± 0.029^de^	4.917 ± 0.029^def^
750	5.467 ± 0.029^ab^	5.033 ± 0.058^d^	4.983 ± 0.029^de^
1000	5.400 ± 0.001^ab^	5.067 ± 0.058^d^	5.100 ± 0.001^cd^
1250	5.300 ± 0.001^bc^	5.100 ± 0.001^cd^	5.100 ± 0.001^cd^

Homogeneous groups (a–f) were created with the use of ANOVA and Tukey’s range test at *p* = 0.01; ±standard deviation

**Fig. 2:**
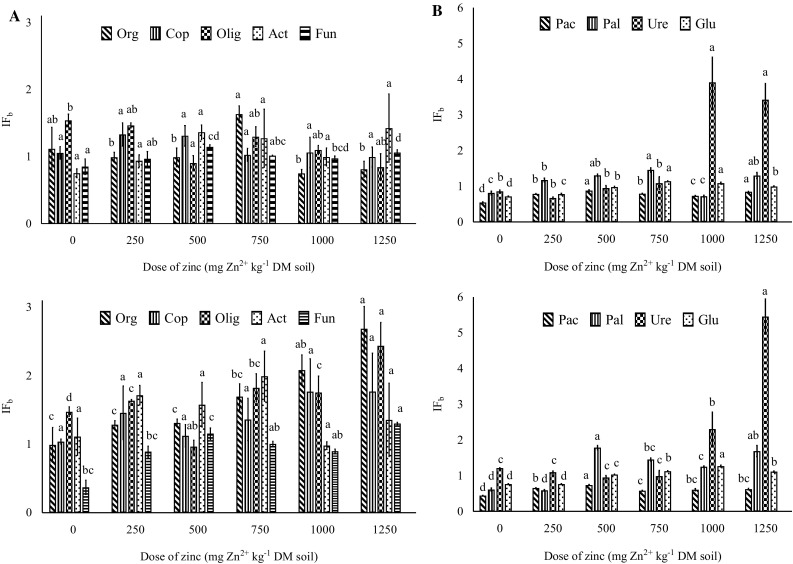
Influence of zinc on the biostimulation index (IF_b_) of microbial growth and enzyme activity in soil fertilized with urea (**A** 250 mg N kg^−1^; **B** 500 mg N kg^−1^). The results were expressed as means of the values noted on two analytical dates (2 and 20 weeks of incubation). IF_b_ = 1 nitrogen has no effect on microbial growth or hydrolase activity, IF_b_ < 1 nitrogen inhibits microbial growth and hydrolase activity, IF_b_ > 1 nitrogen stimulates microbial growth and hydrolase activity. Homogeneous groups (*a–d*) were created separately for microorganisms, enzymes and nitrogen doses (ANOVA and Tukey’s range test at *p* = 0.01). *Zn* ^*2+*^ dose of zinc, *Org* organotrophic bacteria, *Cop* copiotrophic bacteria, *Olig* oligotrophic bacteria, *Act* actinomycetes, *Fun* fungi, *Pac* acid phosphatase, *Pal* alkaline phosphatase, *Ure* urease, *Glu* β-glucosidase

Nitrogen’s ability to modify the structure of microbial communities in soil has been widely discussed in the literature (Stark et al. 2007; Wang et al. 2015; Yu et al. 2015). The values of the biostimulation index (Fig. 2) suggest that nitrogen exerts protective effects on microorganisms in soil contaminated with zinc. All microbial groups proliferated at a higher rate in soil fertilized with urea. The applied doses of zinc and nitrogen also played an important role. Zinc had a stronger stimulatory effect on microbial growth in treatments exposed to the highest dose of urea than in treatments fertilized with 250 mg N kg^−1^. Our findings indicate that nitrogen exerts a protective effect on microbial proliferation in zinc-contaminated soil, which influences the activity of soil enzymes. The enzyme biostimulation index provides information about urea’s neutralizing effects on the activity of alkaline phosphatase, urease and β-glucosidase in zinc-contaminated soil.

The degree of enzyme stimulation was determined by the nitrogen dose and zinc concentrations in soil, which was particularly noticeable in urease activity in treatments exposed to the highest zinc doses (1000 and 1250 mg Zn^2+^ kg^−1^ soil DM). Other authors also demonstrated that nitrogen fertilization increased the activity of β-glucosidase (Saiya-Cork et al. 2002), urease and acid phosphatase (Saiya-Cork et al. 2002; Wang et al. 2008). Nitrogen stimulates soil microorganisms which produce more soil enzymes when biogenic elements become more available. In a study by Jiao et al. (2011), C, N and P concentrations in soil were correlated with phosphatase activity, but not with urease activity. The above results indicate that enzymes respond differently to various soil biochemical parameters.

The results of this and other studies suggest that enzyme activity is conditioned by various overlapping factors, including physicochemical parameters, exposure to toxic compounds and the influence of stimulating and protective substances. In this experiment, zinc had a stimulatory effect when applied in smaller doses, but higher doses of this element strongly inhibited the activity of soil enzymes. Nitrogen’s influence on enzyme activity was also determined by the applied dose of urea. The protective effect exerted by nitrogen on enzymes varied subject to changes in soil acidity. The responses of the analyzed hydrolases to different doses of zinc and nitrogen varied over time.
